# PrimerPooler: automated primer pooling to prepare library for targeted sequencing 

**DOI:** 10.1093/biomethods/bpx006

**Published:** 2017-05-12

**Authors:** Silas S. Brown, Yun-Wen Chen, Ming Wang, Alexandra Clipson, Eguzkine Ochoa, Ming-Qing Du

**Affiliations:** 1 University of Cambridge Computer Laboratory, 15 JJ Thompson Avenue, Cambridge, CB3 0FD, UK; 2 Department of Pathology, Division of Cellular and Molecular Pathology, University of Cambridge, CB2 0QQ, UK

**Keywords:** multiplex PCR, bit patterns, PCR-based next-generation sequencing

## Abstract

Targeted next-generation sequencing based on PCR amplification involves pooling of hundreds to thousands of primers, for preamplification and subsequent parallel single/multiplex PCR. It is often necessary to allocate the set of primers into subpools, a common issue being potential cross-hybridization. For smaller numbers of primers, pool division can be done manually using trial and error to minimize potential hybridization, but this becomes inefficient and time consuming with increasing numbers of primer pairs. We developed PrimerPooler that automates swapping of primer pairs between any user-defined number of subpools to obtain combinations with low-potential interactions. PrimerPooler performs inter-/intra-primer hybridization analysis to identify the adverse interactions, as well as simultaneous mapping of all primers onto a genome sequence in a single run without requiring a prior index of the genome. This allows detection of overlapping primer pairs and allocation of these primer pairs into separate subpools where tiling approaches are used. Using PrimerPooler, 1153 primer pairs were assigned to three preamplification pools (388, 389 and 376 primer pairs each), then 144 subpools of six- to nine-plex PCR for Fluidigm Access Array PCR, followed by Illumina MiSeq sequencing. With optimized experimental protocols, an average of 3269 reads was achieved for the targeted regions, with 95% of targets covered by at least 50 reads, the minimal depth of reads for confident variant calling. PrimerPooler provides a fast and highly efficient stratification of primer pairs for targeted enrichment, thus ensuring representative amplification of the targeted sequences. PrimerPooler is also able to analyse degenerate primers, and is thus also useful for microbiological identification and related target sequencing.

## Introduction

Targeted sequencing of genome regions of interests is a common application in biomedical research. There are now several target enrichment methodologies, including PCR amplification, hybridization capture [1, 2], and selective circularization [3]. PCR amplification-based target enrichment is commonly used when the number of regions to be studied is relatively low, and the DNA sample available is suboptimal in quantity (small tissue or cytology specimen after routine histological diagnosis) or quality (formalin-fixed paraffin-embedded tissue/cytology specimen, circulating tumour DNA). To overcome the low copy number of intact template DNA, a preamplification step is often used with a large number of primer pairs in a single reaction, followed by multiplex PCR to generate sufficient amplicons for next-generation sequencing. Such PCR-based target enrichment is highly flexible at the user end, as various target regions can be freely combined or omitted by choice of PCR primers.

A major challenge in PCR-based enrichment is to ensure that all the targeted regions are representatively amplified, thus uniformly covered following sequencing. This requires careful primer design, ensuring all the primers have similar length, GC content, melting temperature, etc. There are several primer design software packages with some publicly available [4–8], which can be used for stringent primer design. However, pooling a number of primers together in the same reaction invariably creates chances for undesired primer interactions, which adversely impact on their amplification and consequently sequence coverage [9]. Therefore, allocating primers into different preamplification and multiplex PCR reactions to avoid any undesired primer interaction is an important optimization step [9]. Ideally, each preamplification and multiplex PCR will be composed of primer pairs least likely to form any potential undesired interactions. For smaller numbers of primer pairs, pool division can be done manually using trial and error, but this becomes impractical when dealing with large numbers of primer pairs.

MPprimer [10], MultiPLX [11] and Primux [12] can automatically group primers, but these tools have limitations and a low degree of flexibility:

1. The number of subpools generated cannot be defined by the user; they simply create as many pools as necessary to keep the interactions below a preset threshold. This is problematic for methodologies where it is the ‘number’ of subpools, rather than the interaction ‘threshold’, that is fixed.
2. Manual assistance is sometimes required to analyse tiling approaches, which are commonly used to ensure a total coverage of the region to be sequenced. The presence of overlapping primer pairs can lead to the generation of short amplicons, preventing appropriate amplification of the full targeted sequences. Existing tools typically require manual input of which primer pairs overlap, a process that can be time consuming and prone to error.
3. Degenerate primers, which are commonly used in sequence regions harbouring polymorphism or for microbi al identification, are also not compatible with most tools (Table 1).

**Table 1:** Comparison of available tools for constructing libraries for next-generation sequencing

	MPprimer	MultiPLX	PriMUX	PrimerPooler
Length of primers that can be analysed for interaction (bp)	Not defined	Not defined	Not defined	128
Time for interaction analysis of 2000+ primers	Very slow	35 min at 2.3 GHz	Very slow	Fast (seconds)
Able to analyse degenerate primers?	No	No	Yes	Yes
Handling of overlapping primer pairs	Overlaps must be manually specified	None	None	Automatically scans genome file for overlaps
Methods to generate given number of subpools of multiplex PCR	Design multiplex PCR primers subpool by subpool using interactions that are below a set threshold. No absolute control over final number of subpools.			Primer pairs are swapped between a given number of subpools until lowest primer pair interactions is attained

There is not currently a tool which can analyse a large number of designed primer pairs and automatically distribute the primer pairs into a user-specified number of subpools with minimal potential interactions. In this article, we report PrimerPooler that streamlines and automates large-scale primer interaction analysis and primer pool division. The software runs on Windows, Mac and GNU/Linux systems. It supports three steps: inter-/intra-primers hybridization analysis, genome mapping and the automated swapping system. The program itself is quite small, typically compiling to <200 kb of machine code.

**Figure 1:**
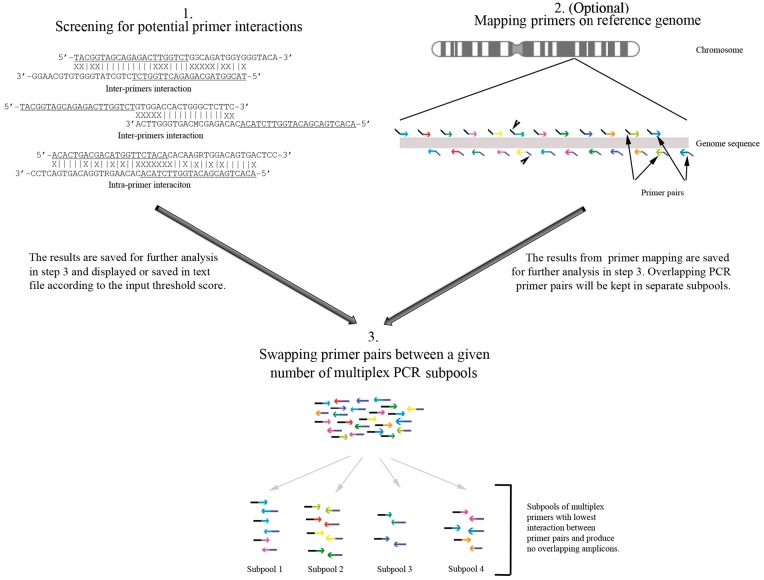
Schematic showing the three parts of PrimerPooler. This includes (1) inter-/intra-primers hybridization analysis, (2) genome mapping and (3) the automated swapping system. For (1), the common sequences for both forward and reverse primers used as indexing/barcoding are underlined. For (2) and (3), the index sequences for primers are represented by black and blue lines attached to the 5′ ends of forward and reverse primers, respectively. The arrow heads in (2) point to the common sequences. Primer pairs are represented by the same colour. The length of each primer is not necessarily shown in proportion in the diagram.

Each step of the program uses new algorithms to achieve fast and accurate performance:

1. The inter-/intra-primers hybridization analysis uses bit-pattern techniques [13] for unprecedented speed.
2. The simultaneous mapping of all primers onto a genome sequence in a single run, without requiring a pre-generated index of that genome, uses a parallel search strategy for quick performance currently considered a challenge. This identifies overlapping primer pairs, which will be placed in separate reactions.
3. The automatic swapping system performs primer swapping according to the results from the first two analyses, using repeated and parallelized iterations of a hill-climbing approach from multiple randomized starting points. The software is able to swap primer pairs between a given number of subpools until low-potential interactions are attained (Fig. 1).

In addition, PrimerPooler is able to handle degenerate primers in all of the above stages.

## Materials and methods

PrimerPooler consists of three steps (Fig. 1). The software takes a text file with a list of primer sequences in FASTA format as input, and outputs FASTA lists of primer pair combinations in a user-defined number of subpools after the three steps of processing.

### Input of data for analyses

PrimerPooler takes a simple text file with multiple primer sequences in FASTA format as the primer list input. The software can recognize a primer pair as part of an amplicon as long as the primers were labelled with the same name apart from the last letter, where F is assumed to stand for the forward and R the reverse primer in each pair.

Common sequences such as barcode or index tags that are to be added at the 5′ end of each forward and reverse primer should be presented as a separate pair of primer sequences in the input text file, named tagF and tagR. These tag sequences will be added automatically to each forward and reverse primer in the list for further hybridization analysis, but not for overlapping analysis.

The maximum length of each primer to be analysed is 128 base pairs (bp), including optional tags at the 5′ end of the primer. The program can recognize upper and lower case A G C T and degenerate bases (R, Y, M, K, S, W, H, B, V, D, N), with or without spaces, in 5′ to 3′ orientation. An example of an input text file is shown in Table 2. Once the text file has been successfully loaded, the software reads the whole file and displays for the user the information on number of primers, the minimum and the maximum length of the primers, the maximum length of the tag sequences and the expected maximum length of the primers with tag sequences in the list.

**Table 2:** Examples of input for PrimerPooler

Example (5′-3′)	Description
>tagF	Common sequence tagged at the 5′end of each forward primer
Acactgacgacatggttctaca	
>tagR	Common sequence tagged at the 5′ end of each reverse primer
tacggtagcagagacttggtct	
>ARID1A_ex1-F	Primer pair of amplicon ‘ARID1A_ex1’ with sequences all in upper case
CGCCGTCTTCCACCAACAA	
>ARID1A_ex1-R	
GGTAGGCGCTGCGGTT	
	
>CIITA_ex5_1-F	Primer pair of amplicon ‘CIITA_ex5_1’ with sequences all in lower case
actcaccttgggctttcatt	
>CIITA_ex5_1-R	
agcaggctttggagtcaa	
	
>CCND3_ex2_1-F	Primer pair of amplicon ‘CCND3_ex2_1’ with sequences in mixed case with spaces
CtY c ag acc cagc agtga	
>CCND3_ex2_1-R	
GATG gTCAGGGGCGTGGT	

### PrimerPooler method

#### Step 1: Hybridization analysis

PrimerPooler begins by analysing inter-/intra-primer interactions for all the user-supplied primers, taking into account the common sequence tagged at the 5′ end of each primer in the list using bit-pattern techniques [13] for unprecedented speed (see ‘Algorithms’ section below for details). Only the highest interaction of each inter-/intra-primer interaction is counted and scored according to the number of nucleotide matches and mismatches (matching score) or according to the estimated thermodynamic value *ΔG* (Gibbs free energy; kcal/mol). Calculated *ΔG* values can vary depending on which nearest-neighbour thermodynamic parameters are used [14–18]; in this program, we adopted data from the source code of MPprimer [10], which was derived from seven publications of Allawi *et al,* e.g. Allawi and SantaLucia [18]. Users are strongly recommended to use *ΔG* rather than simple matching score, to check the stability of complementarity as this is more quantitative and reliable [19].

**Figure 2:**
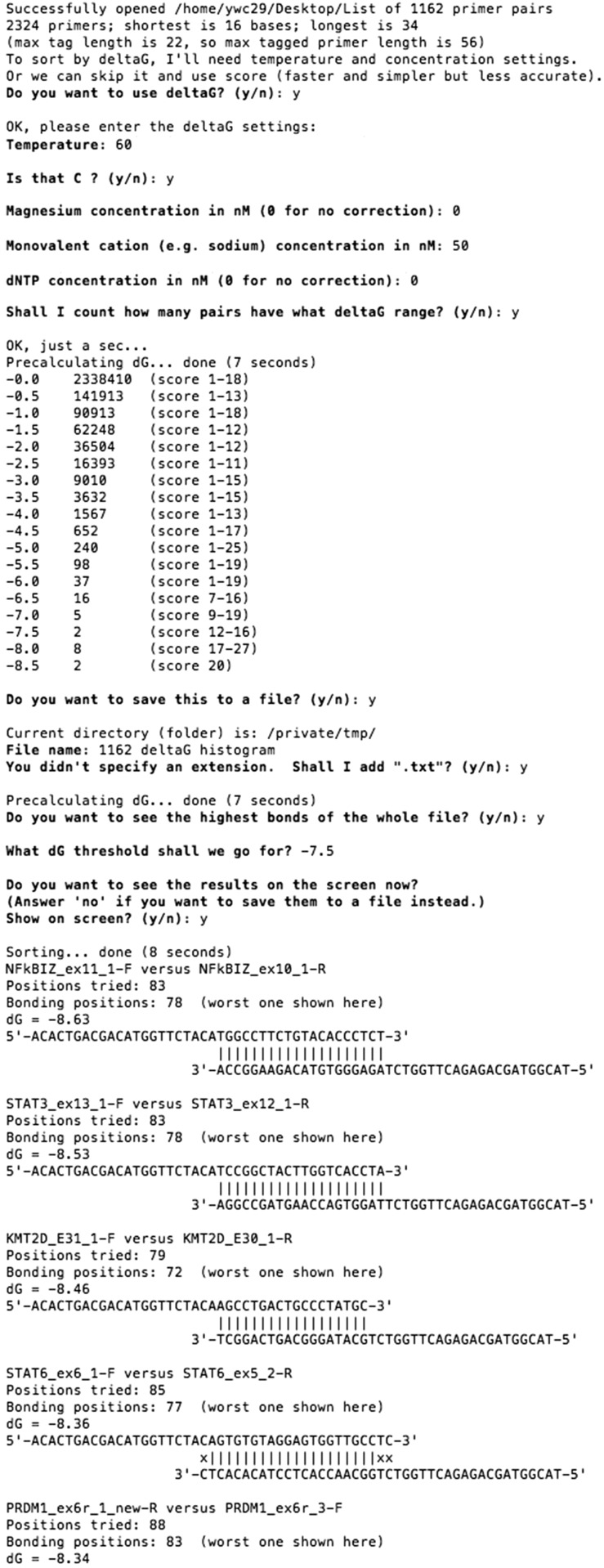
Example screenshot of PrimerPooler after interaction analysis. PrimerPooler sorts and summarizes the number of interactions for each *ΔG* range. The highest bonding among inter- or intra-primer pairs within the primer pool is displayed according to the strength of the interaction. The strongest interactions are displayed first.

After the hybridization analysis, which normally takes seconds or less even for thousands of primers, the program shows a summary of the number of inter-/intra-primer interactions within each matching-score/*ΔG* range, and bonding diagrams of interactions with *ΔG*/score above a user-defined threshold, with the worst interactions being displayed first. The results can be shown on screen and/or saved to a text file. An example screenshot of the program after interaction analysis is shown in Fig. 2.

#### Step 2:***G***enome sequence mapping of primers

PrimerPooler provides the option of mapping a genome file for overlap analysis before splitting the primer pairs into subpools. This option reads a .2bit or FASTA genome file of any species, e.g. from UCSC (http://hgdownload.soe.ucsc.edu/). The program also needs users to enter the maximum length of amplicons (not including the common sequences tagging at the 5′ end of each primer pair) in the primer list for overlap analysis. Incorrect input will lead to non-recognition of any primer pairs that form amplicons with length longer than the maximum input. The genome-overlap search function currently supports a maximum of 32 bases; therefore, the program will look at only the last 32 bases of longer primers. Any primers longer than this will be indicated on screen.

**Figure 3:**
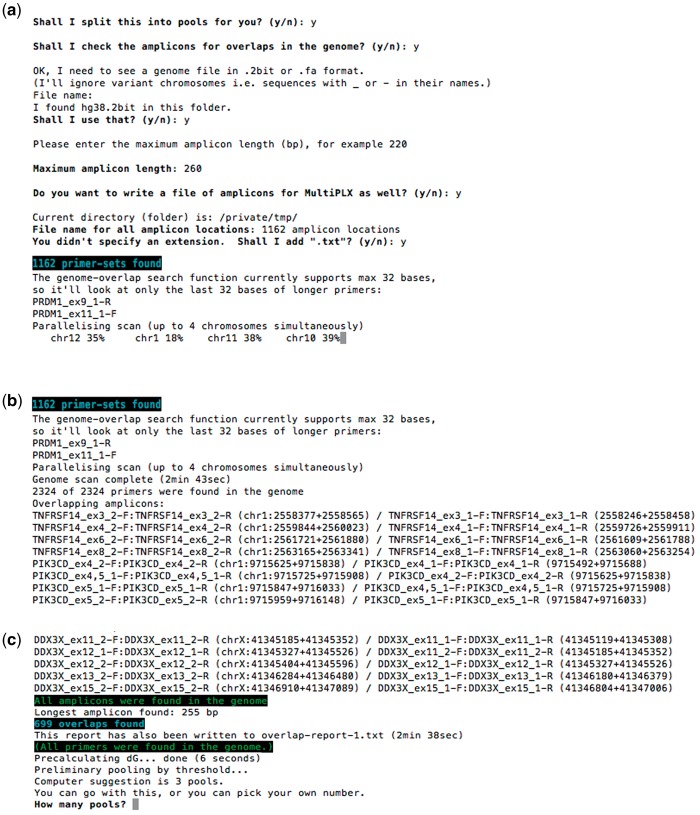
Example screenshot of PrimerPooler for genome mapping of primers. (**a**) Users are asked if they would like to have overlap checks for primer pairs; if so, they should have the reference genome sequence, in 2bit format, downloaded to the computer before the analysis. The progress of the genome mapping of each chromosome are shown in percentages. (**b**, **c**) After the genome mapping analysis of the primers, users can get the information on the time to complete the genome scan, the number of primers input for the anlysis and the list primers that have sequence length longer than 32 bp. Users can obtain reports on overlapping primer pairs with coordinates of the corresponding amplicons.

After the genome mapping and overlap analysis, the program displays to the user the list of primer pairs (with chromosome coordinates) that overlap, and the list of primer pairs (if any) that did not match with any region in the reference genome. The results are displayed on screen and/or saved in text files. Unrecognized pairs are displayed as a warning, as the program is not able to verify that they will not overlap with other pairs in their subpools. An example screenshot of the program after genome scan is shown in Fig. 3.

Mapping of large-scale short sequences onto genome is still considered a great challenge. This part of the program is able to perform simultaneous mapping of thousands of primers onto a genome sequence in a single pass without requiring a precalculated index, and returns the results within a few minutes. The program converts primers into 2bit format and then uses a parallel search strategy for quick performance. The detailed algorithms for genome mapping and overlapping analysis are described in the ‘Algorithms’ section below.

#### Step 3:***D***istribution of primers into subpools

The program swaps primer pairs into a user-defined number of subpools. After being presented with a suggested number of subpools (determined by a single-pass threshold-based packing using a *ΔG* threshold of −7 and avoiding overlaps), the user is asked for their decision on how many pools, and is given the option of setting a maximum size of each pool in order to obtain more even subpools. Swapping is stopped once the program empirically observes that the interactions of each subpool are not being minimized any further, or at the user’s request. During the analysis, the user is able to see the summary of the total number of inter-primer interactions for each score/*ΔG* range and the number of primers in each subpool, for each swap.

**Figure 4:**
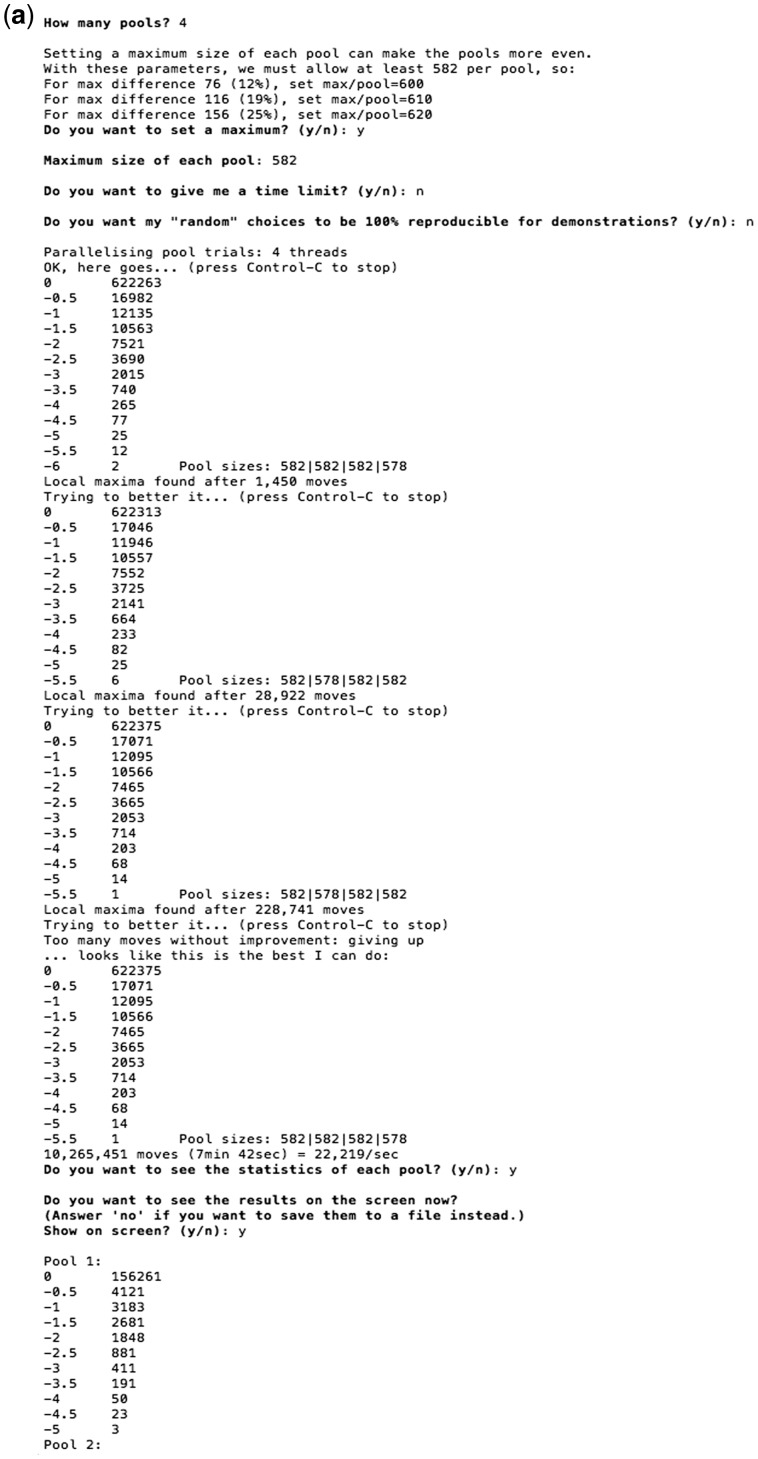
Example screenshot of PrimerPooler for automated distribution of primer pairs. (**a**) Users are asked to input the number of subpools they would like to divide the primer pool into. They are also asked to input the number of primers for each subpool so that they can obtain subpools of primers with similar size, if desired, before the primer swapping. Swapping is stopped once the interactions of each subpool cannot be minimized any further, or at the user’s request. After the analysis, PrimerPooler summarizes the number of interactions in each subpool for matching-score or *ΔG* range and the number of primers in each subpool.

**Figure 5:**
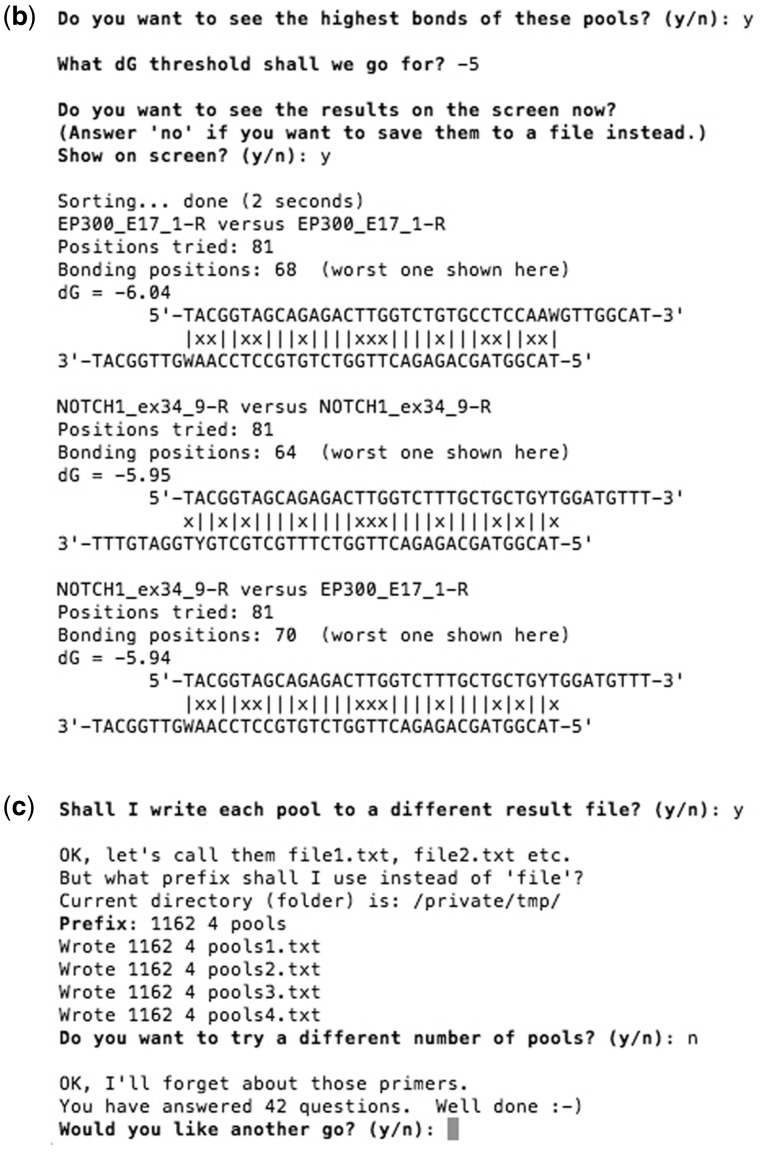
Example screenshot of PrimerPooler for automated distribution of primer pairs. (**b**) Users are able to see the gross bonding results from the pooling and (**c**) have a list of the primer pairs in each subpool written to a file. All timings shown were performed on a Mac with 16 GB RAM and Intel i5 CPU (64-bit, 3.1 GHz).

After the analysis, PrimerPooler displays the best results found. The user is provided with the option to save the result of the list of primer pairs of each subpool to a single text file or to separate files. Example screenshots of the program running the automated primer pairs distribution are shown in Figs 4 and 5.

The systematic evaluation of every possible partitioning scheme is rarely feasible. We used a hill-climbing algorithm from multiple random starting points, and parallelized on multicore CPUs, for quick performance. The algorithms for this step are described in detail in the ‘Algorithms’ section below.

### Access to PrimerPooler

PrimerPooler was written in C and is distributed under the GNU General Public License. The source code and executables are freely downloadable from http://ssb22.user.srcf.net/pooler/.

### Validating PrimerPooler

To examine the performance of the software, we applied two sets of primer data. The first set included 694 sequences (347 PCR primer pairs) from 23 genes. The second set included an additional 815 PCR primer pairs, totaling 2324 primer sequences from 56 genes. Both sets of primers contained overlapping primer pairs. Sequences of the primers for the two sets can be downloaded from our web page listed above. Time running for each part of the program is noted to show the efficiency of the program.

As there is no publicly available integrated software package performing exactly the same as PrimerPooler, we assessed PrimerPooler by comparing the results of its three steps individually with the most commonly used software for each of these steps.

## Algorithms

This is a technical section provided by the corresponding author who wrote the code.

### Fast scoring

Richards [13] showed it is possible to solve Chess board problems in parallel using bit fields: the 64 bits in a 64-bit register can represent the 64 squares of a Chess board. A similar technique can be applied to DNA bases. For example, consider the consider the 2-bit format illustrated here.

It can be seen that two bases will bond if and only if their A|T digits are equal and their G|T digits are not. Since this corresponds to the binary operators XNOR ($\bar{⊕}$) and XOR (⊕), the bonding pattern is given by:

$$\text{bonds}\;:=(p_{A\text{|}T}^{1}\bar{⊕}p_{A\text{|}T}^{2})\wedge (p_{G\text{|}T}^{1}⊕p_{G\text{|}T}^{2})\wedge \text{valid}\_\text{bits}$$

and on a 64-bit CPU, up to 64 bases can be compared simultaneously by this one formula. The resulting number of bonds can then be counted using the ‘popcnt’ (population count) instruction, which is a single instruction on x86 CPUs and on other architectures is trivially implemented using Richards’ technique [13] and it is now a built-in function in the GNU Compiler Collection (GCC). Similarly, various aspects of *ΔG* calculations can be run faster by processing them in parallel using bit-pattern techniques; for full details, please see our source code. Once the bonding pattern is derived at one position, it is normally a matter of simple bit-shifting in a loop to test it at all positions, although if the combined lengths of both primers exceeds the register width then one of them must be reloaded to compensate for the lost binary digits.

If some primers are longer than 64 bases, the inner loop is slowed by the need to manipulate multiple 64-bit registers to contain everything. In 64-bit GCC, the code for this register manipulation can be generated automatically by using GCC’s __uint128_t type, which will work up to 128 bases per primer. Most primers are much shorter than this and can, therefore, be treated with the simpler, faster version of the code that deals with smaller primers.

### Handling degenerate primers

A degenerate primer contains at least one base whose value is uncertain. If one or more primers in an interaction are degenerate, they are represented using four registers instead of three:

R_A:_ bases that might be A

R_C:_ bases that might be C

R_G:_ bases that might be G

R_T:_ bases that might be T

For example, a base G is represented by placing a bit 1 into its corresponding position of R_G_ and 0 in the other three registers; a degenerate base W is represented by placing a 1 into R_A_ and R_T_ and a 0 in the other two. In this system of representation it is no longer necessary to hold a separate validity register, since an invalid position can be represented simply by writing 0 against all four bases. It can be seen that this approach will handle both normal and degenerate primers, but there is value in reserving it for cases where degenerate primers are involved because the bonding computations require more CPU operations:

$$\text{bonds}\;:=(R_{A}^{1}\wedge R_{T}^{2})\vee (R_{C}^{1}\wedge R_{G}^{2})\vee (R_{G}^{1}\wedge R_{C}^{2})\vee (R_{T}^{1}\wedge R_{A}^{2})$$

plus there are more registers to shift each time. Our implementation, therefore, contains data structures to hold primers in either of the above forms, to convert from the first to the second and to use the appropriate version of the bonding computation.

### Genome search and overlap detection

We detect overlapping amplicons by reading the 2bit or FASTA genome file. Each chromosome is shifted, one base at a time, through a single 64-bit register, such that the last 32 bases seen can be read off in 2bit format. If multiple CPU cores are present then these can be used to process multiple chromosomes in parallel (we used GCC’s support of OpenMP for this).

Prior to reading the genome, all primers are converted into 2bit format; if any primers are degenerate, all their possible base combinations are added to the list; additionally, an inverted, complemented version of each primer is added to the list for dealing with the negative strand. This can result in a list of many thousand primer variants. Each of these is limited to its last 64 bits (32 bases), along with a validity mask to track its length. The validity mask of the shortest primer is then used to generate a second 64-bit value for each primer variant, in effect recording each variant’s last *N* bases where *N* is the length of the shortest primer, and this second value is used as a key in a lookup table for a standard binary search. This binary search is then applied at each position of the genome, to answer the question ‘which, if any, of our primer variants have last *N* bases matching the *N* bases we’ve just seen in the genome’. Since the genome is large, it is important that this initial binary search returns failure as soon as possible if no primer is present; since a search can be performed more quickly if the primer length is fixed, the initial search is performed over the second, fixed-length search keys. Only if this initial search returns positive is the entire length of the primer checked to see if there is a true match. If all this searching is performed using 64-bit registers, it can be very fast indeed: the entire genome can be searched for thousands of primers within a few minutes.

To detect amplicon overlaps in the genome, the occurrences of primers that start and end the amplicons are treated as ‘events’ in a hypothetical sequential read through each chromosome. This metaphor allows the code to reason about which amplicons are ‘in progress’ at the ‘time’ a new amplicon starts. However, the ‘amplicon events’ are not actually processed while the genome is being read by the program; they are processed in a separate, simulated pass after the actual reading is complete. This is because the user can set a maximum amplicon length: on encountering an ‘amplicon start’ event, the program needs to look ahead in the event list to find a corresponding end event and verify the amplicon length will fall within the limit before marking that amplicon as ‘in progress’. The program works from an ordered event list somewhat like that used in Gradint [20], and the in-progress array uses two bit fields to distinguish between negative-strand-first and positive-strand-first primer pairs and ensure that the opposite primer orientation is what ends the pair.

Once the program detects that two amplicons overlap, their overlap is reported, and their constituent primers have their mutual interaction scores artificially inflated to an overwhelmingly high value, so that the pool-separation algorithm (below) will immediately separate that pair of amplicons and ensure they stay separate on subsequent moves.

### Pool partitioning

The systematic evaluation of every possible partitioning scheme is rarely feasible. Even with just 100 primers and 2 pools, there are over 10^30^ possible combinations, and even if a billion of these could be evaluated per second on each of a billion computers across the planet, the operation would still take over 30 000 years to complete. It is, therefore, necessary to find a reasonable partitioning without checking every possibility. Thankfully, we find this can be done very quickly by mimicking the way a human might solve the problem.

Initially, each amplicon is allocated a pool at random, and is also given a four-dimensional (4D) ‘pool score’, implemented as four 16-bit fields in a 64-bit integer, to assess the interactions between this amplicon’s primers and every other primer in its pool. The four dimensions of the pool score are:

1. the maximum matching score of any single interaction (if using *ΔG*, the program arbitrarily transforms it as score = int(−2 × *ΔG*) so that it is split into ranges of width 0.5);
2. the number of interactions of that score/range;
3. the number of interactions scoring 1 less than that score/range; and
4. the number of interactions scoring 2 less than that score/range.

All primer pairs’ 4D pool scores are updated the moment any pair is added to or removed from a pool; this can be done very quickly if the list of other primers affected by a pair (and their interaction values) is obtained from a lookup table created using the first part of the program, so that primer interactions do not need repeated recalculation.

In addition to tracking a pool score for each amplicon in its current pool, the program also tracks what its pool score would become if it were moved to each of the other pools. Thus, candidate moves can be ranked according to which move would cause the best overall reduction of pool score, sorting first by the reduction in maximum-score/*ΔG* range, then by the reduction in number of that score/range, then by reduction in number of the one below it, etc.

At each iteration, all primer pairs are searched for the best-ranking move to another pool, and, provided this move provides above-zero benefit, it is performed. This fundamental hill-climbing algorithm can quickly reach a local maxima: a state in which no further improvements can be made on the next move. Since that state is not necessarily the best overall state, it is saved, and then a random number of random ‘bad moves’ are made before starting the iteration again to see if it now converges on a better state. If instead it converges on a worse state, the program re-randomizes the pools and tries again—we found this yielded improvements more quickly than adjusting the number of ‘bad moves’, and it was also more amenable to parallelization on multiple CPU cores, each of which can try a different random starting point in parallel. If no better state can be found within a fixed number of iterations, the previously saved state becomes the final answer. The user may also interrupt the program at any time (or set a timer to do so), at which point it terminates with the best answer it has found so far. Its best answers are usually found within a few minutes; allowing it to run for longer rarely helps.

## Experimental validation

Using the above PrimerPooler, 1153 primer pairs designed to cover 53 genes were assigned to three preamplification pools (388, 389 and 376 primer pairs each) and then 144 subpools of six- to nine-plex PCR for Fluidigm Access Array PCR. The experiments were carried out essentially as described previously with the following modifications [9]. Two different amounts of DNA sample (50 ng and 75 ng) from each specimen were used for preamplification and 1 μl of two different diluted preamplified products (2.5- and 5-fold dilution) were used for Fluidigm Access Array PCR. The PCR product purification, barcoding, library preparation and Illumina MiSeq sequencing were carried out as described previously [9]. The depth coverage and variant calling were also performed as previously described [9].

## Results and discussion

A total of 1153 primer pairs were designed for mutation screening of 53 genes in lymphoma using Fluidigm Access Array PCR and Illumina MiSeq sequencing. We chose three preamplification reactions to enrich the target sequences for Fluidigm PCR based on our experience from a recent study using DNA samples from formalin-fixed, paraffin-embedded (FFPE) tissues [9]. Based on the PrimerPooler analyses, theses primer pairs were divided into three pools (388, 389 and 376 primer pairs each) to avoid any major potential undesired interaction. The Fluidigm 48.48 Access Array allows 48 separate PCRs across 48 DNA samples in parallel per fluidigm IFC (integrated fluidic circuits). To further enhance the power of Fluidigm PCR, multiplex PCR was carried out. In our previous study, based on empirical tests, we were successful to perform four- to six-plex PCR using Fluidigm 48.48 Access Array and achieve adequate coverage following Illumina MiSeq sequencing. However, a further increase of the number of primer pairs for Fluidigm multiplex PCR lead to unbalanced amplification and poor coverage on some of the amplicons. Using PrimerPooler, the 1153 primers were assigned to 144 subpools containing six to nine primer pairs with *ΔG* values weaker than −1.5 at 60°C in 10 min (the user can define the temperature and other parameters, Table 3), corresponding to three Fluidigm Access Array PCR. The running time of the main functions of each step was measured for the two data sets (Table 3).

**Table 3:** Example run times

Number of PCR primers:	694		2306	
Base-match score/*ΔG*:	Score	*ΔG*	Score	*ΔG*
Screening PCR primers and summarizing result counts	1 s	1 s	1 s	3 s
Screening PCR primers and saving the bonding results	1 s @ ≥ 7	2 s @ ≤−7	1 s @ ≥ 7	5 s @ ≤−7
Mapping to human genome and give overlapping results and coordinates for all amplicons	1 min 13 s	1 min 17 s	1 min 26 s	1 min 25 s
Dividing into three pools (without early stop)	2–5 min ∼96k move/s	3–4′ ∼96k	8–15′ ∼22k	10–20′ ∼22k
Dividing into four pools (without early stop)	2–3 min ∼85k move/s	2–5′ ∼87k	10–12′ ∼21k	10–15′ ∼20k
Dividing into	96 subpools^a^		144 subpools^b^	
	1 s ∼5–6k move/s	1–3″ ∼7–8k	3–10″ 4–500	5–40″ ∼600

^a^Time taken when gross inter-primer pair interaction was weaker than score 6 or ΔG −1.5; ^b^weaker than score 6 or *ΔG* −1.0.

**Figure 6:**
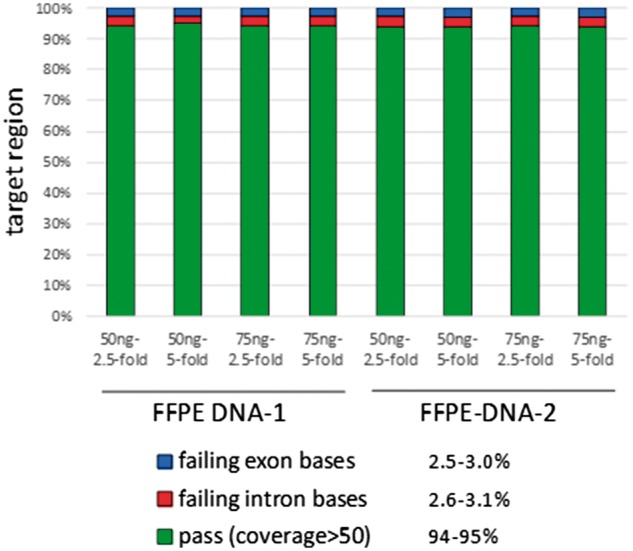
Coverage of targeted sequence regions by Fluidigm Access Array multiplex PCR and Illumina MiSeQ sequencing. Two FFPE DNA samples were tested under various experimental conditions including 50- and 75-ng DNA for pre-amplication, and 1l of 2.5- and 5.0-fold diluted pre-amplified products for Fluidigm Access Array multiplex PCR. An average of 3269 reads was achieved for the targeted regions, with 95% of targets covered by at least 50 reads, the minimal depth of reads for confident variant calling.

Two DNA samples from FFPE tissues were subjected to preamplification, Fluidigm Access Array multiplex PCR and Illumina MiSeQ sequencing based on the primer combination suggested by the above PrimerPooler analyses. The sequencing coverage data are shown in Fig. 6. Under the optimized experimental protocols, an average of 3269 reads was achieved for the targeted regions, with 95% of targets covered by at least 50 reads, the minimal depth of reads for confident variant calling. Based on the two DNA samples tested, both 50ng and 75ng starting amounts of DNA yielded very similar results, and similarly there was no obvious benefit when less diluted pre-amplified product (2.5 versus 5-fold dilution) was used for Fluidigm Access Array multiplex PCR (Fig. 6). The 5% of target regions that were not adequately covered were mainly those with high GC content and difficulty for primer design.

### Hybridization analysis

We assessed PrimerPooler’s interaction analysis by comparing with two of the most commonly used alternatives: AutoDimer [19] (http://www.cstl.nist.gov/div831/strbase/AutoDimerHome page/DownloadPage.htm) and Multiple Primer Analyzer from ThermoFisher Scientific (https://www.thermofisher.com/uk/en/home/brands/thermo-scientific/molecular-biology/molecular-biology-learning-center/molecular-biology-resource-library/the r mo-scientific-web-tools/multiple-primer-analyzer.html; hereafter MPA). Using our smaller data set, the three tools gave the same inter-/intra-primer interactions, albeit with the need to convert degenerate primers into non-degenerate form for AutoDimer. Moreover, it took AutoDimer more than 3 h to show the results of the bondings of the interactions with score threshold of 7, and AutoDimer cannot be used to analyse larger primer sets as it is limited to 1000 sequences per run. MPA was able to show all potential interactions in a few seconds for both primer sets but does not rank them. PrimerPooler is able to process the larger data set, including optional tags at the 5′ of the primer, within a few seconds (Table 3). PrimerPooler also sorts and summarizes the number of interactions for each *ΔG*/score range, with optional bonding diagrams.

### Genome mapping and overlapping analysis

The genome mapping ability of PrimerPooler was compared with the commonly used tool BLAT (https://genome.ucsc.edu/cgi-bin/hgBlat). BLAT can quickly find sequences of 95% and greater similarity of length 25 bases or more. However, it finds perfect sequence matches only if primers are at least 20 bases in length, and can run only 10 primers at a time. PrimerPooler outperformed BLAT by performing the genome mapping of 2306 primer sequences in a single run, taking under 3 min to complete the genome mapping and display the coordinates of the primers and overlap analysis result from scratch (Table 3).

### Swapping of primer pairs

We evaluated the performance of PrimerPooler on pool division ability by comparing with MPprimer [10], MultiPLX [11] and PriMUX [12] (Table 1). PrimerPooler is the only software able to divide the primer sets into a user-specified number of subpools. Swapping is stopped once the interactions of each subpool are not being minimized any further, or at the user’s request. After the analysis, PrimerPooler summarizes the number of interactions for each matching-score/*ΔG* range and the number of primers in each subpool. A list of the primer pairs in each subpool can then be written to a file.

A score threshold of seven or eight works well when designing multiplex PCR primers [21]. MPprimer uses a stringent cutoff value of −7 kcal/mol when grouping primer pairs into subgroups [10]. When using PrimerPooler to swap 1153 primer pairs into three subpools, the highest *ΔG* value for inter-primer pair interaction among the three subpools was −6 kcal/mol, when the temperature was set at 60 °C and the Na^+^ was at 50 mM. PrimerPooler distributed the primers into a user-defined number of subpools with the interaction stability score less than that produced by other primer grouping programs.

In summary, PrimerPooler provides an innovative solution to the problem of primer interactions in large multiplex reactions. The software, together with an optimized experimental protocol, offers a reliable and efficient way to choose and combine a large number of primers for PCR-based target enrichment, ensuring representative amplification and sequencing coverage.
